# In Vitro Anti-*Candida albicans* Mode of Action of *Enterococcus mundtii* and *Enterococcus faecium*

**DOI:** 10.3390/microorganisms11030602

**Published:** 2023-02-27

**Authors:** Svetoslav Dimitrov Todorov, Richard Weeks, Igor Popov, Bernadette Dora Gombossy de Melo Franco, Michael Leonidas Chikindas

**Affiliations:** 1 ProBacLab, Laboratório de Microbiologia de Alimentos, Departamento de Alimentos e Nutrição Experimental, Faculdade de Ciências Farmacêuticas, Universidade de São Paulo, São Paulo 05508-000, SP, Brazil; 2 Food Research Center (FoRC), Departamento de Alimentos e Nutrição Experimental, Faculdade de Ciências Farmacêuticas, Universidade de São Paulo, São Paulo 05508-000, SP, Brazil; 3 Health Promoting Naturals Laboratory, School of Environmental and Biological Sciences, Rutgers State University, 65 Dudley Road, New Brunswick, NJ 08901, USA; 4 Center for Agrobiotechnology, Don State Technical University, Gagarin Square 1, Rostov-on-Don 344002, Russia; 5 Department of General Hygiene, I.M. Sechenov First Moscow Medical University, Bolshaya Pirogovskaya Str., 19/1, Moscow 119146, Russia

**Keywords:** *Enterococcus* spp., *Candida albicans*, bacteriocins, BLIS, probiotics

## Abstract

*Candida albicans* is an important vaginosis causative agent, affecting several women worldwide each year. This study reports on two strains of lactic acid bacteria (*Enterococcus mundtii* CRL35 and *Enterococcus faecium* ST88Ch) expressing bacteriocin-like inhibitor substances (BLIS) active against *C. albicans* 1281. Both strains were γ-hemolytic and not affected by numerous antibiotics, contraceptives, and commercial drugs, suggesting safety for human use. The recorded antimicrobial activity of semi-purified BLIS was 25,600 AU/mL for *E. mundtii* CRL35 and 800 AU/mL for *E. faecium* ST88Ch. Treatment of BLIS with 1 mg/mL proteinase K resulted in complete loss of antimicrobial activity against *Listeria monocytogenes* ATCC 15313 and partial loss of activity against *C. albicans* 1281. The killing effect of the semi-purified BLIS on cell suspensions of *C. albicans* 1281 after 9 h of contact was dose-dependent: for *E. mundtii* CRL35, 400 AU/mL to 25,600 AU/mL caused 63.61% to 79.35% lysis, while for *E. faecium* ST88Ch, 200 AU/mL to 800 AU/mL caused 29.32% to 31.25% cell lysis. The effects of temperature, pH, and presence of the contraceptive Nordette-28 on the adsorption levels of the BLIS to *C. albicans* 1281 were also evaluated. Nordette-28 (10% or 20%) promoted increased adsorption of both studied BLIS to the cells of *C. albicans* 1281 at pH 5.0, while a minor effect was observed at pH 3.0. Different levels of aggregation between *C. albicans* 1281 and *E. mundtii* CRL35 or *E. faecium* ST88Ch were recorded, and optimal adsorption levels were recorded at 37 °C. Appropriate BLIS-producing strains can effectively contribute to the equilibrium of vaginal microbial *status quo* and reduce negative consequences from the development of *C. albicans* infections.

## 1. Introduction

Representatives of lactic acid bacteria (LAB), including *Enterococcus* spp., are natural inhabitants of the urogenital area (UGA) of humans and other animals [1]. Enterococci in the UGA can play beneficial and/or harmful roles in a strain-specific manner. Some strains of *Enterococcus faecalis* were previously reported as associated with urinary tract infections [2,3], especially in HIV-infected patients [2]. Moreover, some representatives of *Enterococcus* spp. were also linked to secondary bacteremia in patients [4]. Several strains of *Candida albicans* have been identified as infectious agents in 80–90% of reported cases of *Candida*-associated vaginosis [5], affecting more than one billion women worldwide each year [6]. Approximately half of those affected experience recurrent infections [7]. Inadequately treated or untreated urogenital infections (UGI) may lead to additional secondary clinical complications, including kidney infection (pyelonephritis), impaired renal function, complications related to pregnancy such as premature delivery, infertility, and fetal mortality, pelvic inflammatory diseases, cervical cancer, and even increased transmission of HIV [8,9,10,11,12].

Pathogenic microorganisms, including those associated with urological clinical complications, can form biofilms, and colonize a targeted ecological niche in the host, thus enhancing the microbial community’s survival levels and tolerance to antibiotic treatment [13,14,15,16]. According to recommendations from the Centers for Disease Control and Prevention (CDC), uncomplicated vaginal candidiasis can be treated with a short course of topical formulations using over-the-counter intravaginal agents, such as clotrimazole, miconazole, and tioconazole, or with prescription intravaginal agents, such as butoconazole, terconazole, and fluconazole. In 80–90% of cases, symptoms and cultures become negative in three days (https://www.cdc.gov/std/treatment-guidelines/candidoasis.htm, accessed on 1 February 2023). When complications appear, antibiotic therapy may be required.

Generally, physicians will prescribe antibiotic treatments in addition to azole containing intravaginal creams [17]. However, extensive antibiotic treatment can negatively influence the healthy commensal microbiota and may lead to a decrease in the natural population of LAB in the vagina, which may in turn result in the colonization of the environment by vaginal pathogens, with an increase in the acquisition of sexually transmitted diseases [18,19], bacterial vaginosis [20,21,22] and UGI [23].

From a microbial balance point of view, vaginal infections can be treated by re-colonizing (restoring) the UGA with LAB, which are an integral part of the natural microbiome present in the vagina [6]. If carefully selected and correctly administered, these bacterial strains may adhere to mucus or epithelial cells and play a primary role in forming a physical barrier against invasive pathogens [24]. These strains can produce a variety of antimicrobial metabolites, such as hydrogen peroxide, organic acids, and antimicrobial peptides (bacteriocins or bacteriocins-like inhibitory substances) [25,26]. It has been suggested that LAB, including different strains of *Enterococcus* spp., can play beneficial roles as probiotics and contribute to human and other animals’ health [21,23,26]. Moreover, the production of bacteriocins/BLIS can be an additional beneficial characteristic of these probiotic strains and contribute to the health-promoting properties of the probiotics and in combating relevant pathogens [21,23,26].

Contraceptives, such as hormonal and non-hormonal intrauterine devices, can influence the vaginal microbiota and increase the risk of developing acute or recurrent vaginal infections: they can facilitate colonization by *Candida* spp. [27] and increase susceptibility to sexually transmitted infections, including herpes simplex virus (HSV), human papilloma virus (HPV), and HIV [28]. Moreover, negative consequences of the application of different contraceptives have been reported, including increased risks of vaginal dysbiosis, a decrease of lactobacilli, and a reduction of natural defenses, facilitating an increase in the numbers of some pathogens—all of which were reported along with the relevant impact of the applied drugs on the immune response [28].

This study aimed to screen for LAB with activity against *C. albicans* activity and explore their killing effect under simplified and favorable in vitro microbial growth conditions at a specific pH and in the presence of contraceptive agents.

## 2. Materials and Methods

### 2.1. Strains and Growth Conditions

LAB evaluated in this study as producers of antimicrobial metabolites or as control test organisms in the following experiments were cultured in MRS broth (Difco, Franklin Lakes, NJ, USA) [29]. *C. albicans* cultures were grown in Sabouraud broth (yeast extract, 10 g/L; peptone, 20 g/L; glucose, 20 g/L; all from Difco) [30]. Incubation of all strains was at 37 °C in aerobic conditions, without agitation, for 24 h. Microbial (bacterial and *C. albicans*) stocks were stored at −80 °C in the presence of 40% (*v/v*) glycerol. One specific vaginal human isolate of *C albicans, C. albicans* 1281, was selected as a reference microorganism based on its sensitivity to antimicrobials.

### 2.2. Screening of LAB for Anti-Candida Activity

A total of 75 LAB strains, isolated from plants, animals, fermented food products, and humans (culture collections, USP, Sao Paulo, SP, Brazil and CERELA, Tucuman, Argentina) belonging to the species *Lactiplantibacillus plantarum*, *Lactiplantibacillus paraplantarum*, *Lactiplantibacillus pentosus*, *Lacticaseibacillus paracasei*, *Lacticaseibacillus casei*, *Limosilactobacillus fermentum*, *Latilactobacillus curvatus*, *Latilactobacillus sakei*, *Pediococcus pentosaceus*, *Pediococcus acidilactici*, *Enterococcus faecium*, *E. faecalis*, *Enterococcus hirae*, *Enterococcus mundtii*, *Enterococcus casseliflavus, Leuconostoc mesenteroides*, *Leuconostoc lactis*, *Lactococcus lactis*, *Streptococcus thermophilus*, and *Streptocococus infantarius* subsp. *infantarius* were evaluated for the production of metabolites with activity against clinically associated *C. albicans* strains (used in the preliminary screening process for selection of appropriate test microorganisms for further studies) by the agar-spot test method [29]. The tests were performed by spotting 10 μL of cell-free supernatants (CFS) of the LAB strains onto Sabouraud medium supplemented with 1% agar (Difco, *w*/*v*) plates and seeded individually with different *C. albicans* strains (10^6^ CFU/mL, final concentration). Antimicrobial activity was expressed as arbitrary units (AU) per mL, taking into consideration the highest serial two-fold dilution showing a clear zone of growth inhibition and the volume of spotted antimicrobial material and using the following formula:

$$\mathrm{AU}/\mathrm{mL}\;=\;\frac{D^{n}\;\times \;1000}{p}$$

where *D* is the type of serial dilution, *n* represents the highest dilution where at least 3 mm of inhibition was recorded, *p* is the volume of spotted BLIS containing material in μL and 1000 is the conversion factor between μL and mL [29].

Inhibition due to H_2_O_2_ production was ruled out by culturing the LAB strains on MRS agar plates supplemented with 0.25 mg/mL 3′,3′-5′,5′-tetramethylbenzidine (TMB) (Sigma-Aldrich, St. Louis, MO, USA) and 0.01 mg/mL horseradish peroxidase (Roche Diagnostics, Basel, Switzerland). The presence of blue colonies indicated H_2_O_2_ production [24].

### 2.3. Purification of the Antimicrobial Metabolites (BLIS) Produced by the Selected LAB Strains

For the partial purification of the produced by studied LAB antimicrobial proteins, recommendations from Bughaloo-Vial et al. [31], Song et al. [32], and Surovtsev et al. [33] were followed. Two LAB strains selected in the screening tests for activity against *C. albicans* 1281 (*E. mundtii* CRL35 and *E. faecium* ST88Ch) were cultured in 500 mL MRS broth for 24 h at 37 °C. CFS were collected via centrifugation (5000× *g*, 10 min, 4 °C), and the pH was corrected to 6.0–6.5 with sterile 1 M NaOH, and then heat treated for 10 min at 80 °C to prevent the lytic effect of potentially produced extracellular proteases. Proteins were precipitated from the CFS with 70% saturated ammonium sulphate. Precipitates were collected after centrifugation (10,000× *g*, 1 h, 4 °C) and suspended in 10 mL 25 mM ammonium acetate buffer (pH 6.5). The obtained suspensions were dialyzed against 5 L of sterile distilled water using a SpectraPor^®^ membrane with a 1000 Da cut-off (Spectrum Inc., Anaheim, CA, USA). In the next steps, suspensions containing antimicrobial peptides were separated on a SepPakC18 hydrophobicity chromatography column by step gradients of 20, 40, 60 and 80% isopropanol in 25 mM ammonium acetate buffer (pH 6.5). At all steps of the purification process, samples were collected and tested for activity against *C. albicans* 1281 and *L. monocytogenes* ATCC 15313. As controls, 20, 40, 60, and 80% isopropanol in 25 mM ammonium acetate buffer (pH 6.5) solutions and pure buffer were tested for potential antimicrobial activity against *C. albicans* 1281 and *L. monocytogenes* ATCC 15313.

### 2.4. Effect of Proteinase K on the Activity of Antimicrobial Metabolites (BLIS) Produced by the Selected LAB

CFS obtained from *E. mundtii* CRL35 and *E. faecium* ST88Ch as described above were treated for 10 min at 80 °C. Each sample of heat-treated CFS was separated into two equal portions: one was treated with proteinase K (1 mg/mL) for 2 h at 37 °C, while the second aliquot was used as a control. To stop enzymatic reactions, samples were heated to 98 °C for 5 min [34]. Residual antimicrobial activity against *L. monocytogenes* ATCC 15313 and *C. albicans* 1281 was evaluated as previously described.

### 2.5. Evaluation of the Hemolytic Activity of the Selected LAB

Hemolytic activity was evaluated according to the recommendations of Fugaban et al. [35] and Reuben et al. [36]. Aliquots of overnight cultures of the selected LAB propagated in MRS broth at 37 °C for 24 h were streaked on Trypticase Soy Agar (TSA) (Difco) supplemented with 5% defibrinated sheep blood (*v*/*v*), and plates were incubated at 37 °C for 24 h. The positive control for β-hemolytic activity (associated with the destruction of red blood cells and formation of distinct halos around the colonies) was *Bacillus cereus* ATCC 27348. *Streptococcus pneumoniae* ATCC 49619 served as a positive control for α-hemolytic activity (partial reduction of red blood cells and formation of green or brown halos around the colonies). *Lb. plantarum* ATCC 14917 was used as a positive control for γ-hemolytic activity (lack of hemolysis and no discoloration around the colonies). All three experiments were performed in triplicate on two independent occasions.

### 2.6. Aggregation Properties of E. mundtii CRL35 and E. faecium ST88Ch

**Figure 1:**
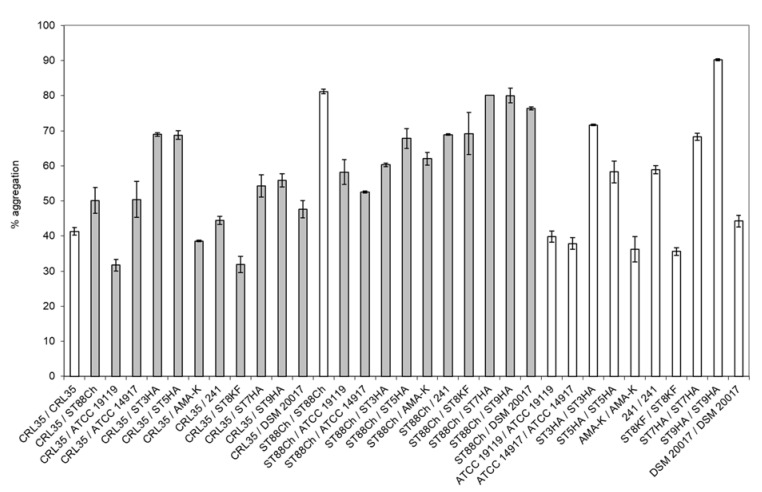
Aggregation (auto-aggregation (white bars) and co-aggregation (grey bars)) properties between CRL35: *E. mundtii* CRL35; ST88Ch: *E. faecium* ST88Ch; ATCC 19119: *L. ivanovii* subsp. *ivanovii* ATCC 19119; ATCC 14917: *Lb. plantarum* ATCC 14917; ST3HA: *P. pentosaceus* ST3HA; ST5HA: *E. faecium* ST5HA; AMA-K: *Lb. plantarum* AMA-K; 241: *Lb. curvatus* 241; ST8KF: *Lb. plantarum* ST8KF; ST7HA: *Enterococcus* spp. ST7HA; ST9HA: *Pediococcus* spp. ST9HA; DSM 20017: *Lb. sakei* subsp. *sakei* DSM20017. The results represent the average of three independent experiments, and SD are included.

Previously applied experimental approaches for the evaluation of aggregation (auto- and co-aggregation) properties suggested by Pingitore et al. [29] and Collado et al. [37] were adapted for current experiments. *E. mundtii* CRL35 and *E. faecium* ST88Ch were evaluated for their auto-aggregation and co-aggregation properties. For co-aggregation partners, strains with pathogenic and beneficial properties (listed in Figure 1) were selected from the culture collection of USP, Sao Paulo, SP, Brazil. The cultures were grown in 10 mL MRS broth (Difco) or brain heart infusion (BHI broth; Difco) for 24 h at 37 °C without agitation, and cells were harvested by centrifugation (7000× *g*, 10 min, 4 °C). The cells were washed twice, resuspended in sterile saline (0.85% NaCl, *w*/*v*), and the density of the suspension was adjusted to OD_660 nm_ = 0.3.

The efficiency of auto-aggregation was calculated according to Pingitore et al. [29]:

$$\%\;\mathrm{Auto}-\mathrm{aggregation}\;=\;\frac{OD_{0}-OD_{60}}{OD_{0}}\;\times \;100,$$

where *OD*_0_ represents the initial optical density of the suspension, measured at 660 nm. *OD*_60_ refers to the optical density of the upper fraction recorded after the cells were incubated for 60 min at 37 °C and centrifuged for 2 min at 300× *g.*

Co-aggregation properties were determined using equal volumes of paired cell suspensions, prepared as described before and after incubation for 60 min at 37 °C. *OD*_660 nm_ was determined for the upper phase after separation by centrifugation for 2 min at 300× *g* [29]. The efficiency of co-aggregation was calculated according to Pingitore et al. [29] as:

$$\%\;\mathrm{Co}-\mathrm{aggregation}\;=\;\frac{OD_{0}-OD_{60}}{OD_{0}}\;\times \;100,$$

where *OD*_0_ represents the initial optical density of the suspension, measured at 660 nm. *OD*_60_ refers to the optical density of the upper fraction recorded after the cells were incubated for 60 min at 37 °C and centrifuged for 2 min at 300× *g.*

The experiments were conducted twice, each time in triplicate.

In addition, auto- and co-aggregation between *E. mundtii* CRL35, *E. faecium* ST88Ch, and *C. albicans* 1281 in sterile saline (0.85% NaCl, *w*/*v*) were determined after 60 min at 37 °C, at pH 3.0, 5.0, and 7.0, with the aim of partially simulating the growing conditions of a healthy and a *C. albicans*-infected vaginal environment, both in the absence or presence of 10% or 20% (*m*/*v*) Nordette-28 contraceptive (Teva Pharmaceuticals, Basel, Switzerland).

### 2.7. Adsorption of BLIS to C. albicans 1281

Adsorption of the semi-purified BLIS produced by *E. mundtii* CRL35 and *E. faecium* ST88Ch to *C. albicans* 1281 was tested according to Pingitore et al. [29] and Agaliya and Jeevaratnam [38]. *C. albicans* 1281 was grown in Sabouraud broth for 18 h at 37 °C without agitation, and cells were harvested by centrifugation (8000× *g*, 15 min, 4 °C). The obtained cells were washed with sterile 5 mM phosphate buffer (pH 6.5) two times and resuspended in the same buffer to an OD_600 nm_ equal to 1.0. The obtained suspension was mixed with an equal volume of semi-purified BLIS (corresponding to the 60% isopropanol fraction obtained from chromatography on SepPakC18) prepared as previously described from CRL35 or ST88Ch (with 25,600 AU/mL and 800 AU/mL, pH 6.5, respectively) and incubated for 1 h at 37 °C. After removal of the cells (centrifugation at 8000× *g*, 15 min, 25 °C), the antimicrobial activity against *C. albicans* 1281 of unbound extracellular BLIS produced by CRL35 and ST88Ch in the supernatants was estimated as previously described. All experiments were performed in duplicate at minimum.

The efficiency of BLIS adsorption to the target cells was estimated according to the following formula:

$$\%\;\mathrm{adsorption}\;=\;[100\;-\;\frac{\mathrm{AU}/mL_{1}}{\mathrm{AU}/mL_{0}}]\;\times \;100,$$

where AU/mL_1_ represents the BLIS activity after treatment, and AU/mL_0_ the original (before treatment) BLIS activity.

The effect of temperature and pH on BLIS adsorption to *C. albicans* 1281 was estimated as described above, with incubation for 1 h at 30, 37, and 45 °C, and at pH 3.0, 5.0, and 7.0, simulating the changes of vaginal pH in *Candida*-associated infections [17]. CFS were obtained (8000× *g*, 15 min, 25 °C), and the pH was adjusted to 6.0 with sterile 1 M NaOH. BLIS activity in the supernatant against *C. albicans* 1281 was determined as previously described. The experiments were performed at least in duplicate.

#### Effect of the Contraceptive Nordette-28 on Adsorption of the Studied BLIS to *C. albicans* 1281

Cell suspensions of *C. albicans* obtained as previously described were supplemented with 10% (*w*/*v*) and 20% (*w*/*v*) Nordette-28. The pH of cell suspensions was adjusted to 3.0, 5.0 and 7.0 with 1 M NaOH or 1 M HCl. The studied BLIS were added to the treated cell suspensions, as described previously, and incubated for 1 h at 37 °C without agitation. The CFS were obtained by centrifugation (8000× *g*, 15 min, 25 °C) and the activity of the BLIS were determined as previously described against *C. albicans*. The experiments were performed in at least in duplicate.

### 2.8. Effect of Antibiotics and Generic Drugs, including Selected Contraceptives, on the Growth of E. mundtii CRL35 and E. faecium ST88Ch

Experimental approaches for the evaluation of the effect of commercial non-antibiotic drugs and antibiotics on growth of LAB previously suggested by Todorov et al. [39] and Amaral et al. [40] were applied. Overnight cultures of the two enterococcal strains were embedded into BHI supplemented with 1% (*w*/*v*) agar to a final concentration of approximately 10^6^ CFU/mL. Antibiotic disks, listed in Table 1, were placed on the agar surface, and the plates were incubated for 24 h at 37 °C. The effect of antibiotics was recorded by measuring the diameter of the growth inhibition zones, expressed in millimeters.

In a similar experimental set-up, the effect of selected medicaments applied in specific concentrations, summarized in Table 2, and the commercially available contraceptives Nordette-28, Triphasil (Pfizer, Sydney, Australia), Diphasil (Bayer, Germany), and MicroVal (Saint-Just-Malmont, France) on the growth of *E. mundtii* CRL35 and *E. faecium* ST88Ch were determined. For the listed contraceptives (Nordette-28, Triphasil, Diphasil and MicroVal), tablets numbered 1, 8, 15 and 22 were dissolved in 5 mL of sterile distilled water. An amount of 15 μL of each commercial drug solution was spotted on the surface of BHI agar plates containing *E. mundtii* CRL35 or *E. faecium* ST88Ch (approximately 10^6^ CFU/mL) and incubated for 24 h at 37 °C. Growth inhibition was recorded by measuring the diameter of the zones.

### 2.9. Cell Lysis of C. albicans by BLIS from E. mundtii CRL35 and E. faecium ST88Ch

An experimental approach previously suggested by Todorov and Dicks [41] and Pingitore et al. [29] for the evaluation of the cell lysis of target strains by the effect of antimicrobial agents was applied. Cell lysis experiments were performed using sterile flat-bottom 96-well microtiter plates (Nunc, Sigma Aldrich, St. Luis, MO, USA). *C. albicans* 1281 was cultured in 20 mL Sabouraud broth for 18 h at 37 °C without agitation, and the cells harvested by centrifugation (8000× *g*, 10 min, 4 °C), and washed twice and resuspended in 10 mL of potassium phosphate buffer (20 mM, pH 6.5). A yeast suspension (100 μL) was placed in the microtiter plate wells and supplemented with 50 μL semi-purified BLIS of CRL35 or ST88Ch (corresponding to the 60% isopropanol fraction obtained from chromatography on SepPakC18) solubilized in potassium phosphate buffer (25 mM, pH 6.5) at different serial two-fold dilutions. Microtiter plates were incubated for 24 h at 37 °C and at selected intervals, the absorbance at 655 nm was measured spectrophotometrically (Thermo Fisher Scientific, Waltham, MA, USA). Levels of cell lysis of *C. albicans* 1281, expressed as %, were calculated as [100 − (At/Ao × 100)], where Ao and At were absorbance measured at 0 and 3, 6, 9 or 24 h of incubation, respectively [29].

### 2.10. Statistical Analysis

IBM SPSS 26.0 (SPSS, Inc., Chicago, IL, USA) was used for data analysis. Data were not normally distributed according to the Shapiro–Wilk test. The Kruskal-Wallis test, followed by the Dunn-Bonferroni post-hoc test, was used to evaluate differences among results of obtained experimental data. Results were presented as mean ± standard deviation (SD). The statistical significance was determined as *p* < 0.05.

## 3. Results

### 3.1. Screening of LAB for Anti-Candida Activity

From a total of 75 tested LAB, the CFS (pH 6.0–5.5) of only two strains, *E. mundtii* CRL35 and *E. faecium* ST88Ch, which were previously isolated, identified, and characterized as bacteriocins producers [29], presented inhibitory activity against *C. albicans* 1281, a clinical isolate (6400 AU/mL and 800 AU/mL, respectively). This and all other tests were performed with planktonic *C. albicans* 1281 cells. However, when the CFS was submitted to ammonium sulphate precipitation and elution with the 60% isopropanol fraction after SepPakC18 hydrophobic chromatography, the activity increased to 12,800 AU/mL and 25,600 AU/mL for BLIS produced by *E. mundtii* CRL35, and remained unchanged for BLIS produced by *E. faecium* ST88Ch. It is important to emphasize that the same BLIS activity was recorded after evaluation of the 60% isopropanol fraction that was lyophilized and resuspended in 25 mM phosphate buffer (pH 6.5) as the initial volume. At the same time, neither the controls, 40%, 60% and 80% isopropanol solutions, nor the applied buffer, showed inhibitory properties against *C. albicans* 1281 or *L. monocytogenes* ATCC 15313. The 40% and 80% isopropanol fractions after SepPakC18 chromatography presented lower activity against *C. albicans* 1281—1600 AU/mL and 800 AU/mL for *E. mundtii* CRL35, and 400 AU/mL and 200 AU/mL for *E. faecium* ST88Ch, respectively.

*E. mundtii* CRL35 and *E. faecium* ST88Ch did not produce H_2_O_2_ when grown on modified MRS agar.

Treatment of the CFS of *E. mundtii* CRL35 and *E. faecium* ST88Ch with 1 mg/mL proteinase K at pH 6.0–6.5 resulted in complete loss of BLIS activity when tested against *L. monocytogenes* ATCC 15313. However, when *C. albicans* 1281 was used as a test organism, a low level of inhibitory effect was still observed. This is an indication that both LAB strains (*E. mundtii* CRL35 and *E. faecium* ST88Ch) are BLIS producers. However, some other non-proteinaceous antimicrobial(s) may also be present in the CFS with activity against *C. albicans* 1281.

### 3.2. Hemolytic Activity

*E. mundtii* CRL35 and *E. faecium* ST88Ch were evaluated as γ-hemolytic since no discoloration around the colonies was observed. The bacterial growth of *E. mundtii* CRL35 and *E. faecium* ST88Ch in the performed test was similar to that observed for *Lb. plantarum* ATCC 14917, a γ-hemolytic control.

### 3.3. Aggregation Properties

**Figure 2:**
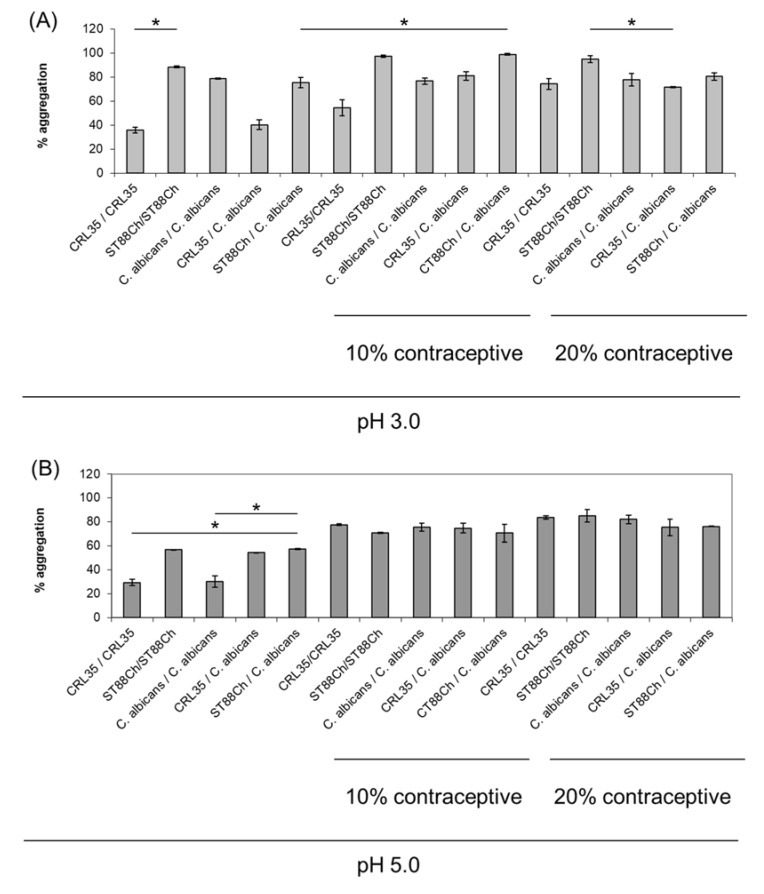

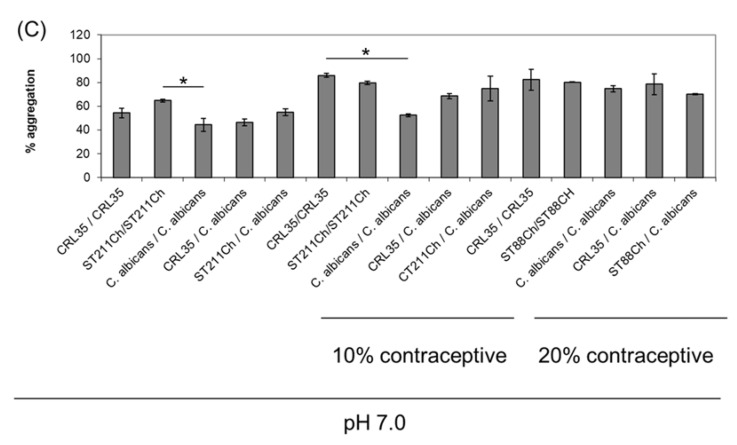
Aggregation properties between CRL35 (*E. mundtii* CRL35), ST88Ch (*E. faecium* ST88Ch) and *C. albicans* 1281 in presence of Nordette-28 (contraceptive) at 10% and 20% and at pH 3.0 (**A**), 5.0 (**B**) and 7.0 (**C**). Aggregation between the two BLIS-producing enterococci and *C. albicans* 1281 was evaluated in the presence of 10 and 20% Nordette-28 (contraceptive) and at a pH representing simplified models of the healthy and infected vaginal environments. Results are the average of three independent experiments, and SD are included. * *p* < 0.05.

Different levels of aggregation were recorded between *C. albicans* 1281 and *E. mundtii* CRL35 or *E. faecium* ST88Ch, and additional strains were utilized as co-aggregation partners in a strain-specific manner (Figure 1). High levels of co-aggregation between *E. mundtii* CRL35 and *E. faecium* ST88Ch were recorded with *C. albicans* 1281 (Figure 2), compared to the other test organisms. This improved co-aggregation can be regarded as evidence of a predisposition for better antimicrobial effect of *E. mundtii* CRL35 and *E. faecium* ST88Ch against *C. albicans* 1281.

### 3.4. Adsorption of BLIS to C. albicans

In the present study, we evaluated the effects of temperature, pH, and the presence of contraceptives (Nordette-28) on the adsorption of the studied BLIS to *C. albicans* 1281 cells. The experimental conditions were selected to provide a simplified version of the favorable growth conditions of a healthy vaginal environment (37 °C and mildly acidic pH), and conditions when affected by the presence of *Candida* spp., where the pH was decreased. For the purpose of comparing the adsorption of the studied BLIS to *C. albicans* 1281, increased and decreased temperatures (40 °C and 30 °C) were also applied. Moreover, the presence of the contraceptive was considered a factor that may influence BLIS adsorption. Temperature, pH, and the presence of the contraceptive Nordette-28 affected the adsorption of the studied BLIS to cells of *C. albicans* 1281 (Table 3). Interestingly, at higher pH, representing conditions resulting from increased levels of *C. albicans* in the vaginal environment, the adsorption of both BLIS was higher (Table 3). Optimal adsorption levels were recorded at 37 °C, compared to the evaluated temperature controls of 30 °C and 40 °C.

The presence of 10% or 20% Nordette-28 promoted increased adsorption of both studied BLIS to the cells of *C. albicans* 1281 at pH 5.0 (Table 3). A minor effect was observed at pH 3.0, where only supplementation with 20% Nordette-28 increased adsorption of BLIS produced by *E. mundtii* CRL35. At pH 7.0, adsorption of BLIS produced by *E. mundtii* CRL35 was slightly reduced, while the adsorption of BLIS produced by *E. faecium* ST88Ch was not affected at neutral pH (Table 3).

### 3.5. Effect of Antibiotics and Other Medicaments, including Selected Contraceptives, on the Growth of E. mundtii CRL35 and E. faecium ST88Ch

The two BLIS-producing strains showed moderate resistance to some of the evaluated antibiotics in the present study (Table 1). None of the contraceptives evaluated in this study (Nordette-28, Triphasil, Diphasil, MicroVal) inhibited the growth of *E. mundtii* CRL35 or *E. faecium* ST88Ch. Moreover, additional drugs evaluated as potential inhibitors of the two studied strains showed no antagonism. Only thioridazine (neuroleptic) and Voltaren (anti-inflammatory and anti-rheumatic) showed moderate inhibitory effect, and a slightly stronger inhibition by Diuretidin (diuretic) on *E. mundtii* CRL35 and *E. faecium* ST88Ch was also observed (Table 2).

### 3.6. Cell Lysis of C. albicans by BLIS from E. mundtii CRL35 and E. faecium ST88Ch

**Figure 3:**
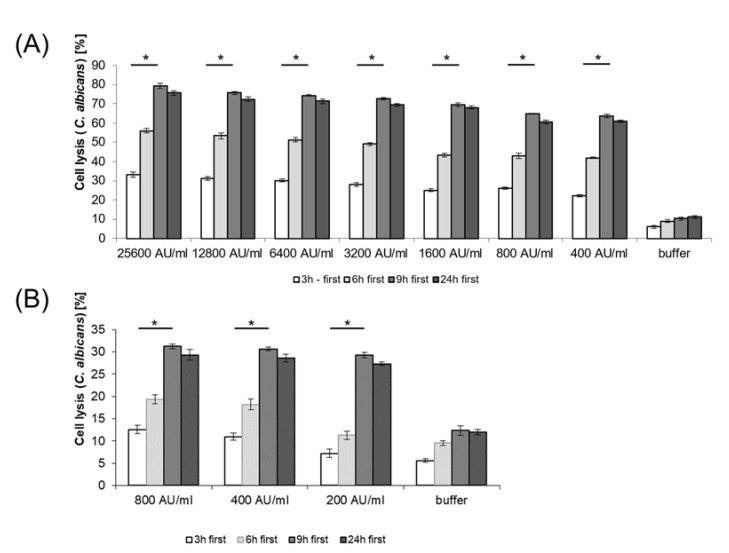
Cell lysis (%) of *C. albicans* 1281 evaluated after 3, 6, 9 and 24h incubation time, in presence of semi-purified BLIS-produced *E. mundtii* CRL35 applied from 400 AU/mL to 25,600 AU/mL (**A**) and semi-purified BLIS-produced *E. faecium* ST88Ch applied from 400 AU/mL to 800 AU/mL (**B**), illustrating the effect of different concentrations of the studied BLIS on killing of *C. albicans* 1281 and the role of contact time between the studied antimicrobials and evaluated test microorganism. Results are the average of three independent experiments, and SD are included. * *p* < 0.05.

The application of semi-purified BLIS from *E. mundtii* CRL35 and *E. faecium* ST88Ch on cell suspensions of *C. albicans* 1281 showed a dose-dependent killing effect (Figure 3). When applied from 400 AU/mL to 25,600 AU/mL, the BLIS produced by *E. mundtii* CRL35 caused 63.61% and 79.35% lysis of *C. albicans* 1281 cells after 9 h of contact (Figure 3A). After 24 h of contact, similar results were observed, with a slight decline of cell lysis (Figure 3A). However, when BLIS produced by *E. faecium* ST88Ch was applied in concentrations from 200 AU/mL to 800 AU/mL, levels of cell lysis of *C. albicans* 1281 were between 29.32% and 31.25%, respectively, after 9 h of contacts (Figure 3B).

## 4. Discussion

BLIS production is a characteristic that is considered to be an additional advantageous property for beneficial therapeutical strains [42]. BLIS such as antimicrobial peptides play an essential role in microbial interactions [43]. In a previous study, *E. mundtii* CRL35 and *E. faecium* ST88Ch were reported as BLIS-producing strains that play an important role in the biopreservation and control of *Listeria monocytogenes* in fresh cheese [29]. In the present study, these two strains, in addition to other LAB strains previously described as producers of antimicrobial peptides and/or presenting probiotic properties and belonging to the culture collections of USP (Brazil) and CERELA (Argentina), were explored for their antimicrobial effect against strains of *C. albicans* isolated from patients diagnosed with vaginosis and positively identified by API 20 C AUX fermentation reactions according to the recommendations of Singh et al. [17]. The majority of the evaluated strains showed no inhibitory activity against the tested *C. albicans* strains assessed in the preliminary screening process for the selection of BLIS producer/s and an appropriate sensitive strain (data not shown), even though most of them presented BLIS activity against *L. monocytogenes* ATCC 15313. The aim of the performed screening process was to select BLIS producers from previously identified bacteriocinogenic LAB in the culture collections of USP and CERELA with unusual anti-*Candida* activity. Taking into consideration the specificity of this task, the screening was also focused on selecting *Candida* spp. that would be sensitive to the BLIS of the LAB candidates. Thus, the screening process needed to be regarded as a dual-task challenge for the selection of the optimal combination of BLIS producer/s and sensitive organisms. Despite the current knowledge that BLIS produced by LAB are more active against genetically related bacterial species than other types of microorganisms [44], the BLIS produced by *E. mundtii* CRL35 and *E. faecium* ST88Ch were active against *C. albicans*. The proteinaceous nature of the expressed antimicrobials was confirmed based on the treatment of the CFS obtained from *E. mundtii* CRL35 and *E. faecium* ST88Ch with proteolytic enzymes at pH adjusted to 6.0. This experimental approach was previously reported as a simple, but reliable, test for confirming the proteinaceous nature of LAB-expressed antimicrobial metabolites [29,34,35]. The fact that a loss of antimicrobial activity was recorded when *L. monocytogenes* ATCC 15313 was applied as test microorganism supports the idea that the expressed antimicrobial metabolite is of proteinaceous nature. However, when *C. albicans* 1281 was used as a test organism in the same experiment, a low residual activity was recorded. This indicates that an additional antimicrobial compound that is different from previously recorded and evaluated bacteriocins produced by *E. mundtii* CRL35 and *E. faecium* ST88Ch [29] may be produced and play a role in the observed unusual anti-*Candida* activity. The fact that antagonistic tests were performed with CFS that was corrected to a neutral pH is an additional piece of evidence showing that the observed inhibitory activity cannot be associated with the direct effects of produced organic acids. Moreover, the specific nature of this additional antimicrobial agent produced by *E. mundtii* CRL35 and *E. faecium* ST88Ch merits a deeper investigation in order to answer the question of whether the observed activity is a consequence of a single mode of action or the result of synergetic interactions together with bacteriocins produced by the two enterococci [29]. Despite being considered unusual [45], Hefzy et al. [46] also reported that BLIS produced by *Lb. pentosus*, *Lb. paracasei* subsp. *paracasei*, *Lactobacillus delbrueckii* subsp. *lactis* and *Streptococcus uberis* strains were active against *C. albicans*. Pentocin TV35b, a bacteriocin produced by *L. pentosus* TV35b, was reported as having activity against *C. albicans* [47]. The results given by the controls (lactic acid solution at pH 4.0, CFS treated with proteinase K, and CFS untreated with proteolytic enzymes) indicated that the observed inhibitory effects are likely due to an antimicrobial compound of proteinaceous nature. Antimicrobials involved in killing and/or inhibitory effects against *C. albicans* can be BLIS or acids or combinations of them. Nieminen et al. [48] reported that D,L-2-hydroxyisocaproic acid (HICA) is an active substance associated with the inhibition of planktonic cells of *C. albicans* and biofilm formation in vitro. Fugaban et al. [26] evaluated the production of different organic compounds, including organic acids, as inhibitors of the growth of filamentous fungal species and suggested that bacteriocins may only have a secondary effect. Ishijima et al. [49] suggested that BLIS produced by *Streptococcus salivarius* K12 can be actively involved in the growth control of *C. albicans*, as shown in an oral candidiasis model. Lum et al. [50] suggested the use of KABT-AMP (lysine-rich peptide) and the antimicrobial peptide Uperin 3.6 from the *Uperoleia mjobergii* frog as potential templates for the development of effective anti-*Candida* peptides. The proposed hybrid peptides, containing a mixed backbone of KABT-AMP and Uperin 3.6, showed potent anti-*Candida* activity with an estimated MIC of around 8–16 mg/L. Scillato et al. [51] reported on the probiotic potential of the vaginal isolates *Lactobacillus gasseri* 1A-TV, *Lb*. *fermentum* 18A-TV, and *Lb*. *crispatus* 35A-TV, including antimicrobial effects of their metabolites in CFS with activity against *C*. *albicans* and *C*. *glabrata.* These authors evaluated the probiotic properties of the studied LAB, including their co-aggregation, adhesion to HeLa cells, and biofilm formation properties. Moreover, *Lb*. *gasseri* 1A-TV and *Lb*. *crispatus* 35A-TV were shown to harbor genes for the production of the *helveticin* J and *acidocin* A bacteriocins. Salari et al. [52] evaluated the antifungal effects of *Lb. acidophilus* and *Lb. plantarum* (cells and CFS) on different *Candida* species, including co-aggregation properties and antimicrobial activity. *Lb. acidophilus* and *Lb. plantarum* in the range of 10^2^ to 10^10^ CFU/mL or as CFS showed inhibitory effects against the evaluated *Candida* species.

*E. mundtii* CRL35 and *E. faecium* ST88Ch did not produce H_2_O_2_. Hydrogen peroxide-producing LAB were suggested to be associated with the antimicrobial properties of the vaginal environment and are involved in spermicidal effects, such as those reported for drugs containing nonoxynol-9 [53]. Different spermicides are used as contraceptive drugs and are involved in the protection of the urogenital tract of women against sexually transmitted pathogens such as *Neisseria gonorrhoea, Chlamydia trachomatis*, and HIV [54]. To be effective probiotics for vaginal health, *E. mundtii* CRL35 and *E. faecium* ST88Ch need to be resistant to different contraceptives and spermicides and thus should be verified as such in appropriate in vivo experiments.

The relationship between gastrointestinal tract (GIT) and vaginal health was previously reported, and oral administration of a combination of *Lacticaseibacillus rhamnosus* GR-1 and *Lb. fermentum* RC-14 was shown to be effective in the treatment of bacterial vaginosis and supported restorations of the urogenital tract microbiota to normal levels. Reid et al. [6,55,56] reported that *Lb. rhamnosus* GR-1 and *Lb. fermentum* RC-14 were able to adhere to uroepithelial and vaginal cells and were actively involved in the inhibition of growth and adhesion of urogenital pathogens. *Lb. rhamnosus* GR-1 was not associated with the production of therapeutic levels of H_2_O_2_ and was resistant to different spermicide drugs, but on the other hand, *Lb. fermentum* RC-14, evaluated as a producer of H_2_O_2_, was sensitive to the effect of tested spermicides [57]. The potential application of *E. mundtii* CRL35 or *E. faecium* ST88Ch as putative urogenital probiotics will probably be more effective if they are taken in combination with H_2_O_2_-producing LAB, and a resulting assortment of different antimicrobial agents may also have synergistic activity.

Aggregation of *E. mundtii* CRL35 and *E. faecium* ST88Ch to *C. albicans* may also be regarded as a valid tool for the reduction of *C. albicans* adherence to epithelial cells. Aggregation between enterococci and *C. albicans* can be considered the primary step in the killing process of *C. albicans*, as a consequence of the close contact between the microbial cells and facilitated delivery of antimicrobial peptides to the target cells. The fact that clinical strains of *E. faecalis* and *C. albicans* are frequently found to co-exist in the infected urogenital tract of patients [51,52,58,59] supports the application of *E. mundtii* CRL35 and *E. faecium* ST88Ch, or their BLIS against *C. albicans*.

Aggregation properties between different microbial species needed to be evaluated considering the complexity of the environment due to the potential existence of a variety of different recorded antimicrobial metabolites. The human vaginal microbiome is a complex system where interactions between different species provide health benefits and contribute to the normal reproductive processes of the host. In natural processes, pathogens face the defense mechanisms of the immune system, but microorganisms considered as beneficial for the vaginal environment also play a significant role in maintaining microbial balance and in reducing the development of clinical complications associated with effective or opportunistic pathogens [1,2,6,7]. Aggregation between different microbial species (called co-aggregation) can facilitate biofilm formation and thereby improve the survival of specific microbial species as part of formed biofilm in different ecological environments, including the vagina and other parts of human and other animals’ bodies. From this point of view, high aggregation (auto- or co-aggregation) needs to be considered a beneficial characteristic when recorded for strains with clear positive effects for the host; such is the case for probiotics. However, when pathogens (effective or opportunistic) are involved in the formation of a stable aggregation complex, this needs to be regarded as facilitating their pathogenicity, since they will be protected by the biofilm and are more present in the environment due to their interactions with other aggregation partners. From a different point of view, if antimicrobial interaction is a clearly established fact between co-aggregation partners, then the co-aggregation processes will facilitate this antimicrobial action since it will provide close contact between a producer of antimicrobials and the target cell and increase the efficacity of killing properties of produced BLIS or other antagonistic metabolites. Moreover, environmental conditions (pH, temperature, different chemicals) can influence these aggregation processes, and appropriate evaluation through in vitro preliminary experiments can help to predict the efficacity of suggested antimicrobials of putative probiotics strains in further in vivo or in situ studies, as has been previously shown by Pingitore et al. [29].

One of the major mechanisms of action of bacteriocins and/or BLIS is associated with an interruption of the cellular integrity of the target organisms via pore formation in the cell wall [44,60]. This process may be associated with the adsorption of BLIS to a specific receptor [60] or simply based on affinity and electro-physical interactions [61]. Thus, the adsorption of BLIS to the cell surface of target organisms is considered a key step in their mode of action.

In the present study, we have evaluated the effects of temperature, pH, and the presence of a contraceptive (Nordette-28) on the adsorption of the studied BLIS produced by *E. mundtii* CRL35 and *E. faecium* ST88Ch to cells of *C. albicans* in conditions modeling healthy and candidiasis-associated vaginal conditions. We observed an increase in the adsorption of the studied BLIS produced by *E. mundtii* CRL35 and *E. faecium* ST88Ch to *C. albicans* 1281 in the presence of Nordette-28 at the evaluated pH levels. However, what needs to be further explored is our understanding of how the presence of the contraceptive improves the antimicrobial activity of the studied BLIS, and whether the contraceptive works synergistically with the BLIS or assists its antimicrobial activity in a complementary manner.

Optimal adsorption levels were recorded at 37 °C, compared to the evaluated temperature controls of 30 °C and 40 °C. In vitro tests for evaluating the effects of BLIS are essential in assessing their effectiveness. An in vitro research approach can be a potential indicator of the effectiveness of the bacteriocinogenic strains in future evaluations *in situ*, as has been shown in the study of Pingitore et al. [29], where the bioprotective effect and the control of *L. monocytogenes* in cheeses by the same bacteriocinogenic strains (*E. mundtii* CRL35 and *E. faecium* ST88Ch) was demonstrated.

The presence of the contraceptive Nordette-28 at a final concentration of 10% or 20% increased the adsorption of both studied BLIS to cells of *C. albicans* at pH 5.0 (Table 3), but very little effect was observed at pH 3.0. However, at pH 7.0, adsorption of the BLIS produced by *E. mundtii* CRL35 was slightly reduced and was not affected by adsorption of BLIS produced by *E. faecium* ST88Ch (Table 3). These results are interesting since they indicate that the use of the contraceptive will not significantly affect the prevention or active treatment of vaginal candidiasis cases by the BLIS or BLIS-producing strains.

The safety of *E. mundtii* CRL35 and *E. faecium* ST88Ch must be thoroughly tested using in vivo studies, since their use as beneficial therapeutical cultures for human consumption is still questionable. Some strains of *Enterococcus* spp. (such as representatives of species *E. faecalis*) have been associated with nosocomial infections [62]. Furthermore, some enterococci are resistant to glycopeptides and antibiotics, produce biogenic amines, and exhibit virulence factors [63]. Future in vivo studies will not only evaluate the safety of *E. mundtii* CRL35 and *E. faecium* ST88Ch, but will also provide information on their efficiency and ability to attach to uroepithelial cells [64].

The safety properties of beneficial microbial strains are of utmost importance and the evaluation of antibiotics resistance is considered a basic requirement in the selection of strains for the practical applications of beneficial therapeutical cultures [65]. The two studied BLIS-producing strains showed moderate resistance to some of the evaluated antibiotics (Table 1). In general, the scientific consensus is that beneficial therapeutical cultures and/or probiotics candidates cannot be resistant to clinically applied antibiotics [65], an argument to avoid the spread of antibiotic-resistant genetic determinates. However, Suvorov [66] defended the hypothesis that the spread of antibiotic-resistant genes (and other virulence factors) is an unlikely scenario in natural environmental conditions. On the other hand, we need to consider whether these genetic determinates are associated with bacterial chromosomes or located on plasmids, since this can influence horizontal gene transfer [66]. It is clear that before the two studied strains (*E. mundtii* CRL35 and *E. faecium* ST88Ch) can be recommended for the application of beneficial therapeutical cultures in the control of *C. albicans*-associated vaginal disorders, additional safety evaluations will need to be performed, including an investigation into the presence of specific antibiotics genes and their locations. Only once the appropriate safety assessments have been conducted can *E. mundtii* CRL35 and *E. faecium* ST88Ch be considered for application as life-protective cultures, or a determination made on whether their antimicrobial peptides (postbiotics) will be more suitable for the control of vaginal candidiasis.

Probiotics aimed at vaginal health can be applied orally or as vaginal suppositories. The relationship between GIT balance and general health has been well described [67]. Several arguments are provided in support of the idea that a healthy GIT has a positive effect on different parts of the body, including the vagina [68,69]. The viability of orally delivered probiotics can be influenced not only by stomach acidity but by the presence of bile salts. They are also exposed to the complex environmental condition of the GIT, including the presence of a multitude of different microorganisms, the immunological response of the host, and different drugs associated with episodical or chronic diseases.

None of the contraceptives evaluated in this study inhibited the growth of *E. mundtii* CRL35 or *E. faecium* ST88Ch. Previously, it was reported that the application of contraceptives could influence the ecological balance of the vaginal microbiota and even increase risks of the development of sexually transmitted diseases because of the reduction of natural protections by LAB and its influence on immune defense [28]. Moreover, it will be interesting for further studies to evaluate the effects of the investigated contraceptives on both the immune response and the vaginal microbiota, including the effect on *E. mundtii* CRL35 or *E. faecium* ST88Ch as determined using an appropriate animal model. Furthermore, most of the generic drugs evaluated as potential inhibitors of the two studied strains cannot be considered antagonists. Only thioridazine, Voltaren, and Diuretidin affected the growth of *E. mundtii* CRL35 or *E. faecium* ST88Ch (Table 2). The inhibitory effect of episodically or permanently applied drugs on the viability of probiotic strains needs to be evaluated and considered to determine the suitability of their combined applications. It was previously shown that several painkillers and drugs used for the treatment of chronic diseases can have an inhibitory effect on some LAB [70,71]. Such interactions merit deeper evaluation and the construction of an appropriate database to avoid negative interactions between generic drugs and probiotics, and optimize both effects.

The effect of most bacteriocins and/or BLIS on test microorganisms is associated with the destabilization of the cell wall, pore formation, and, consequently, bacterial lysis [60]. Application of semi-purified BLIS produced by *E. mundtii* CRL35 and *E. faecium* ST88Ch on cell suspension of *C. albicans* showed a dose-dependent effect in the killing of test organisms (Figure 3). The observed differences between the effect of BLIS produced by *E. mundtii* CRL35 and *E. faecium* ST88Ch on the lysis of *C. albicans* were expected and are in agreement with observed levels of adsorption between the studied BLIS and applied test microorganism (Table 3). Thus, the results agree with previously reported similar experiments evaluating the effectiveness of BLIS produced by *E. mundtii* CRL35 and *E. faecium* ST88Ch against *L. monocytogenes* ATCC 15313 and their possible application in biopreservation processes of fresh cheeses [29]. Appropriate in vitro experiments can serve as an indicator for the selection of appropriate strains for further in situ or in vivo evaluation of their effectiveness as putative probiotics strains.

## 5. Conclusions

Beneficial microorganisms with therapeutical properties (probiotics) can be regarded as effective alternatives for reducing the use of chemical and designed drugs, as some are associated with several negative side effects. Appropriate probiotic microorganisms effectively modulate the vaginal microbiome and reduce negative consequences due to the development of yeast infections (including *C. albicans*). Probiotics with therapeutical properties can interfere with pathogens via cell-to-cell interaction or via the production of several antimicrobial metabolites, such as BLIS, including the establishment of cumulative or synergetic interactions between different factors. Moreover, the application of contraceptives can modulate the beneficial properties of beneficial microorganisms with therapeutical properties and reinforce their antimicrobial effects.

## Figures and Tables

**Table 1:** Effect of selected antibiotics on the growth of *E. mundtii* CRL35 and *E. faecium* ST88Ch. Results are expressed as the size of inhibition zones in millimeters.

Antibioitcs (μg per Disk)	CRL35	ST88Ch
Nitrofurantoin (300)	30	22
Ciprofloxacin (5)	24	20
Fusidic acid (10)	25	20
Furazolidone (10)	21	12
Rifampicin (30)	30	25
Tetracycline (30)	38	38
Ofloxacin (5)	16	16
Cephazolin (30)	13	15
Ceftriaxone (30)	0	18
Erithromicin (15)	23	0
Chloramphenicol (30)	33	30
Vancomycin (30)	24	0
Sulphamethoxazole/trimethorpin (23.75 and 1.25)	17	0
Trimethoprim (5)	16	12
Nalidixic acid (30), Neomycin (10), Tobramycin (10), Cefuroxime (30), Clindamycin (2), Cefotaxime (30), Oxacillin (1), Compound sulphonamides (300), Cefepime (30), Amikacin (30), Ceftazidim (30), Streptomycin (10), Metronidazole (50)	0	0

**Table 2:** Effect of commercial drugs on bacterial growth of *Enterococcus mundtii* CRL35 and *Enterococcus faecium* ST88Ch.

Commercial Name	Original	Final Concentration	Composition (Active Substance)	Medication Form	Producer	Applied Quantity of Drugs (10 μL) and Diameter of the Inhibition Zone (mm)		Medication Group
						CRL35	ST88Ch	
Dimenhydrinat	50 mg	10 mg/mL	Dimenhydrinat 50 mg	Tablets	Pharmacia AD, Bulgaria	0	0	Antihistamine
Dehydratin Neo	25 mg	5 mg/mL	Hydrochlorothiazidin 25 mg	Tablets	Pharmacia AD, Bulgaria	0	0	Moderate diuretic
Famotidine	20 mg	4 mg/mL	Famotidine 20 mg	Film tablets	Medika AD, Sandansky, Bulgaria	0	0	Antiacid
Thioridazin	10 mg	2 mg/mL	Thioridazine hydrochloride 10 mg; starch, colorant E110	Film tablets	Balkanpharma AD, Dupnitza, Bulgaria	5	2	Neuroleptic
Diclamina	70 mg	14 mg/mL	Cinarizina 20 mg; Heptaminol Acetilinato 50 mg	Tablets	Laboratorios Dr. Esteve S.A. Barcelona, Spain	0	0	
Acetylcystein 600 Stada^®^ Tabs	600 mg	120 mg/mL	Acetylcystein 600 mg	Tablets	STADA Arzneimittel AG, Bad Vilder, Germany	0	0	Antitusiva, anti-inflammatory
Duoventrinetten N	500 mg	100 mg/mL	Antazidum 500 mg	Tablets	Pharma Schworer, Wiesenbach, Germany	0	0	
Bisalax	5 mg	1 mg/mL	Bisacodylum 5 mg	Film tablets	Pharmacia AD, Bulgaria	0	0	Treatment of constipation
Diuretidin	37.5 mg	7.5 mg/mL	Triamterenum 25 mg; hydrochlorothiazidum 12.5 mg	Tablets	Pharmacia AD, Bulgaria	12	7	Moderate diuretic
Oleum Jecoris	37.5 mg	7.5 mg/mL	Retinol palmitas (Vit A) 3750 IU Ergocalciferolum (Vit D2) 375 IU Oleum Jecoris Aselli to 37.5 mg	Capsules	TroyaPharma AD, Bulgaria	0	0	Hepatic and pancreatic
Atarax	25 mg	5 mg/mL	Hidroxizina diclorhidrato 25 mg	Tablets	UCB Pharma S.A.	0	0	Nevrolepta, anti-inflammatory
Ambro	100 mg	20 mg/mL	Ambroxol 100 mg	Tablets	Hexal AG	0	0	Anti-inflammatory
Voltaren	50 mg	10 mg/ml	Sodium diclofenac 50 mg	Suppository	Novartis	4	8	Antirheumatic Anti-inflammatory
Proalgin	500 mg	100 mg/mL	Metamizole sodium 500 mg	Tablets	Balkanpharma AD, Dupnitza, Bulgaria	0	0	Analgesic, anti-inflammatory
Novphyllin	100 mg	10 mg/mL	Aminophylline 100 mg	Tablets	Balkanpharma AD, Dupnitza, Bulgaria	0	0	Anti-asthmatic
Cerucal	10.54 mg	2.5 mg/mL	Metoclopramide hydrochloride 10.54 mg	Tablets	Arzneimittelwerk Dresden GmbH, Radebeul, Germany	0	0	Anti-inflammatory
Espumisan (Simethicon)	40 mg	8 mg/mL	Simethicon 40 mg; methyl-4-hydroxybenzoat 0.28 mg	Tablets	Berlin-Chemie (Menarini Group), Berlin, Germany	0	0	Analgesic
Hepcarsil	70 mg	14 mg/mL	Silymarin 70 mg	Capsules	Medica, Sandansky, Bulgaria	0	0	Hepatic and pancreatic

**Table 3:** Evaluation of the adsorption of BLIS produced by *E. mundtii* CRL35 and *E. faecium* ST88Ch to *C. albicans* 1281 at pH 3.0, 5.0 and 7.0 and 30 °C, 37 °C and 40 °C (A), and in presence of 10% and 20% Nordette-28 (B). The presented results are representative of the three repetitions of the mentioned experiments.

**(A)**	**CRL35**	**ST88Ch**
pH 3.0 at 30 °C	33%	50%
pH 3.0 at 37 °C	33%	50%
pH 3.0 at 40 °C	33%	25%
		
pH 5.0 at 30 °C	33%	75%
pH 5.0 at 37 °C	33%	25%
pH 5.0 at 40 °C	33%	25%
		
pH 7.0 at 30 °C	67%	100%
pH 7.0 at 37 °C	83%	75%
pH 7.0 at 40 °C	67%	75%
**(B)**	**CRL35**	**ST88Ch**
37 °C		
pH 3.0	33%	50%
pH 3.0 and 10% contraceptive	33%	50%
pH 3.0 and 20% contraceptive	50%	50%
		
pH 5.0	33%	25%
pH 5.0 and 10% contraceptive	50%	75%
pH 5.0 and 20% contraceptive	50%	50%
		
pH 7.0	83%	75%
pH 7.0 and 10% contraceptive	67%	75%
pH 7.0 and 20% contraceptive	67%	75%

## Data Availability

All data generated by the present project is available upon request.
